# Competition alters seasonal resource selection and promotes use of invasive shrubs by an imperiled native cottontail

**DOI:** 10.1002/ece3.4580

**Published:** 2018-10-25

**Authors:** Amanda E. Cheeseman, Sadie J. Ryan, Christopher M. Whipps, Jonathan B. Cohen

**Affiliations:** ^1^ Department of Environmental and Forest Biology SUNY College of Environmental Science and Forestry Syracuse New York; ^2^ Department of Geography and Emerging Pathogens Institute University of Florida Gainesville Florida

**Keywords:** competition, competitive displacement, early successional forest, eastern cottontail, habitat use, invasive species, New England cottontail, resource selection

## Abstract

Many ecosystems face multiple invaders, and interactions among invasive and native species may complicate conservation efforts for imperiled species. Examination of fine‐scale resource selection can be used to detect patterns in habitat selection resulting from species interactions and assess the value of specific resources, including invasive plants, to wildlife. We used animal location data with mixed‐effects resource selection models to examine seasonal competitive interactions and species‐specific selection for forage and cover resources by an imperiled native lagomorph, the New England cottontail *Sylvilagus transitionalis* and its nonnative competitor, the eastern cottontail *S. floridanus* in the eastern Hudson Valley, NY. We found evidence that resource selection by New England cottontails depended on the relative prevalence of eastern cottontails to New England cottontails. Where eastern cottontails were less prevalent New England cottontail selected for resources characteristic of early successional shrublands. Where eastern cottontails were more prevalent, New England cottontails selected for resources characteristic of later successional shrublands. New England cottontail use of certain invasive shrubs depended on the prevalence of eastern cottontails relative to New England cottontails, suggesting response to invasive plants is confounded by interactions with a nonnative competitor. Our results further emphasize the need for conservation efforts to consider invasive management within the ecosystem context. We demonstrate the utility of resource selection studies to assist in this regard by exploring competitive interactions in the absence of removal studies, while simultaneously assessing the impact of habitat components such as invasive vegetation on species of conservation concern. *Synthesis and applications* Resource selection studies can be directly applied to inform ongoing species conservation where multiple invaders are present or where species interactions influence resource selection. Fine‐scale assessments of resource selection, similar to those presented here, can be used to selectively manage habitat to benefit desired species within the ecosystem context.

## 1 INTRODUCTION

Invasive species are among the top drivers of biodiversity loss and one of the primary challenges to conservation (Pressey, Cabeza, Watts, Cowling, & Wilson, 2007; Wilcove, Rothstein, Dubow, Phillips, & Losos, 1998). Competitive displacement of native species by nonnative competitors and habitat alterations resulting from the proliferation of invasive plants have both led to population declines or extinctions of native species (reviewed in Mooney & Cleland, 2001; Gurevitch & Padilla, 2004; Harris, 2009). In systems with multiple invaders, complex interspecific interactions may hinder or negate the efficacy of traditional management techniques (reviewed in Zavaleta, Hobbs, & Mooney, 2001; Glen et al., 2013; Ballari, Kuebbing, & Nuñez, 2016).

Resource selection studies provide a method to simultaneously assess the value of specific habitat components, including invasive plants, to wildlife and identify niche partitioning or displacement resulting from competitive interactions, without the use of removal (Douglas, Marsh, & Minckley, 1994; Schroeder et al., 2013; Wauters, Gurnell, Martinoli, & Tosi, 2002; Westhoff & Rabeni, 2013). Such studies commonly assume fitness is correlated with fundamental resources provided by habitat, such as food and cover, and the management of these resources might improve not only habitat quality but also fitness within populations (Thomas & Taylor, 2006). Thus, resource selection has become a common tool to identify and prioritize key ecosystem components to conserve when managing declining species (Cole, Jones, & Harris, 2005; Russo, Jones, & Migliozzi, 2002). Where competition is a concern for conservation, such as when one species is declining or nonnative competitors are present, studies of resource selection can help to identify differences in resource use between competing species that can be exploited by managers to benefit a desired competitor, while discouraging use by the undesirable species (Cole et al., 2005; Kenward & Holm, 1993).

Although eradication of invasives is the favored choice for conservation, some invasives act as facilitators to native species by providing or supplementing limited resources, potentially attracting individuals to invasive‐dominated areas (reviewed in Zavaleta et al., 2001; Rodriguez, 2006). In some instances, invasive species have replaced or augmented native resources, becoming necessary for the persistence of endangered species (Van Riel, Jordaens, Martins, & Backeljau, 2000; Zavaleta et al., 2001), and their removal could be detrimental to recovery efforts if appropriate native species are not also restored. Quantifying resource use can illuminate the effect of these invasives on native species to inform invasive management and native species conservation (DeGrandchamp, Garvey, & Colombo, 2008; Recio, Mathieu, Virgós, & Seddon, 2014). A clear understanding of fine‐scale resource selection can inform guidelines to alter competitive interactions and improve habitat at a scale relevant to management. Our goal was to assess whether the prevalence of nonnative eastern cottontails *Sylvilagus floridanus* influenced fine‐scale selection of resources that provide forage and cover to the imperiled New England cottontail *S. transitionalis* within shrublands in eastern New York. As temporal variation in resource availability can alter resource selection patterns, and guidelines developed from resource use over single or pooled seasons may miss seasonally critical habitat components (McCall, Pilfold, Derocher, & Lunn, 2016; Stewart, Bowyer, Kie, Cimon, & Johnson, 2002), we also examined variability in selection by season. We hypothesized that if competitive interactions altered resource accessibility to New England cottontails, we would observe a difference in resource selection by New England cottontails between areas where competitors are more prevalent and where they are less prevalent, and that these differences may vary seasonally as a result of changes in resource availability. We simultaneously assessed selection for abundant invasive plant species, which are of uncertain value for New England cottontails, but are selected for food and cover by eastern cottontails (Morgan & Gates, 1983; Sweetman, 1949). We sought to identify seasonal patterns of resource use that might inform management for the benefit of the imperiled New England cottontail without enhancing populations of the nonnative eastern cottontail. These methods consider ecosystem context to inform management and conservation at a scale relevant to the site‐level habitat management for New England cottontails and can be applied more generally to studies of resource selection between competing species and for species within altered habitats.

## 2 MATERIALS AND METHODS

### 2.1 Study system and species

**Figure 1:**
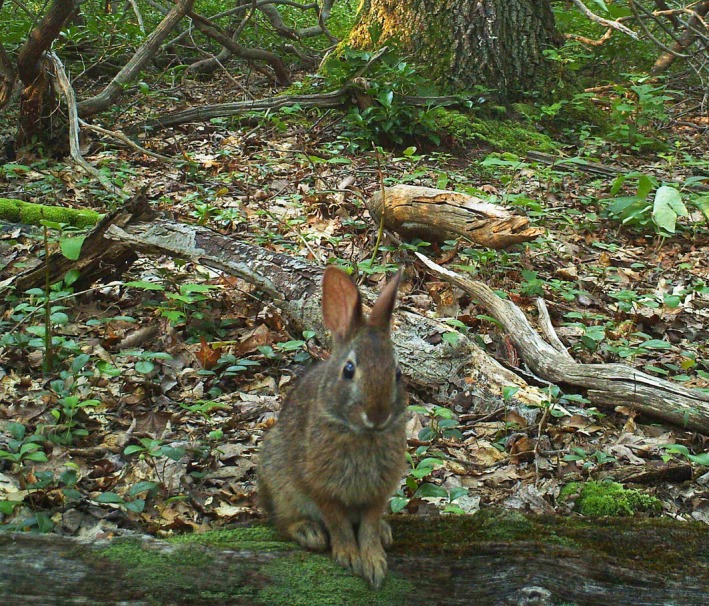
A New England cottontail (*Sylvilagus transitionalis*) in native‐dominated ericaceous shrubland site with an oak (*Quercus* spp.) canopy. Photograph was taken by remotely triggered trail camera.

The shrubland obligate New England cottontail is a lagomorph endemic to New England and eastern New York, USA (Figure 1). The New England cottontail has experienced a range‐wide population decline concurrent with regional losses of successional shrublands (i.e., dense woody shrub communities associated with early successional forest regeneration) over the 20th century resulting from reforestation and human development (Litvaitis, 1993). The species persists within five isolated populations covering less than 14% of its historic range (Fenderson, Kovach, Litvaitis, & Litvaitis, 2011; Litvaitis et al., 2006). Efforts to recover New England cottontails are largely dependent on creating and maintaining a network of suitable shrubland patches (Fuller & Tur, 2012). However, restoration of shrublands to benefit New England cottontails has been complicated by competitive interactions with the nonnative eastern cottontail, the establishment of invasive plants within successional shrublands, and a poor understanding of seasonal resource needs of New England cottontails (Litvaitis et al., 2008).

Native to extreme southern New York and west of the Hudson River, NY, USA (Nelson, 1909), eastern cottontails were introduced east of the Hudson River for hunting in the early 20th century (Foster, Motzkin, Bernardos, & Cardoza, 2002; Probert & Litvaitis, 1996). They have since become widely established, frequently co‐occurring with New England cottontails at the patch scale (Probert & Litvaitis, 1996). Avoidance and antagonistic interactions between New England and eastern cottontails have been recorded in captive trials, but neither species dominated the interactions, suggesting competitive displacement of resident New England cottontails by eastern cottontails was unlikely to occur through antagonistic interactions (Probert & Litvaitis, 1996). However, the eastern cottontail is more general in its habitat requirements and may be able to colonize shrublands at an earlier successional stage than is suitable for New England cottontail occupancy (Probert & Litvaitis, 1996). This system of “prior rights” would confer a colonization advantage to eastern cottontails and limit later colonization or may result in displacement of New England cottontails in co‐occupied successional shrublands (Probert & Litvaitis, 1996). Similarly, displacement from successional shrublands into coniferous forest and ericaceous shrublands has been hypothesized as a driver of the range‐wide decline in Appalachian cottontails (Russell, Moorman, & Guynn, 1999).

There is also concern that common invasive shrubs alter habitat quality for New England cottontails, but their effect on fitness of cottontails is not well understood (Warren, Litvaitis, & Keirstead, 2016). Successional shrublands may be particularly prone to invasion by exotic plant species (Johnson, Litvaitis, Lee, & Frey, 2006), and within Northeastern shrublands, invasive Japanese barberry *Berberis thunbergii* and multiflora rose *Rosa multiflora* are particularly pervasive, causing changes in vegetation structure, and reducing native plant diversity (Silander & Klepeis, 1999; Yurkonis, Meiners, & Wachholder, 2005). Multiflora rose provides forage to cottontail rabbits, and eastern cottontails are known to consume Japanese barberry during winter (Dalke & Sime, 1941; Sweetman, 1949). However, the presence of Japanese barberry reduces the biomass of other plant species (Silander & Klepeis, 1999) and its value as a food plant to New England cottontails is unknown. Further, these invasives are associated with higher tick burdens on New England cottontails, which may negatively impact fitness (Mello, 2018).

Although New England cottontail body condition and survival are sensitive to resource availability (Smith & Litvaitis, 2000; Villafuerte, Litvaitis, & Smith, 1997), seasonal differences in the resource needs of New England cottontails are poorly understood. Prior studies of New England cottontail habitat have been primarily constrained to data collected during winter (Barbour & Litvaitis, 1993; Buffum, McGreevy, Gottfried, Sullivan, & Husband, 2015; Villafuerte et al., 1997), which is a limiting period for survival but does not consider differences in resource availability between summer and winter or variation in the resource needs of juveniles or reproducing individuals during the late spring to summer reproductive season.

### 2.2 Study area

**Figure 2:**
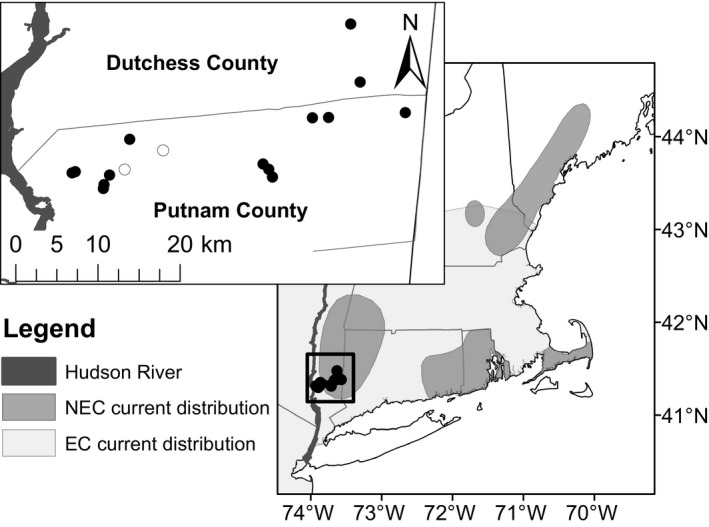
Study sites where New England and eastern cottontails co‐occurred (black circles), and where only New England cottontails were detected (open circles) in New York State, 2013–2016, and IUCN Red List Data (Version 2.1, Cambridge, United Kingdom) ranges of New England (*Sylvilagus transitionalis*, NEC) and eastern cottontails (*S. floridanus*, EC).

We studied New England and eastern cottontail resource selection from December 2013 to July 2016 at sites of known New England cottontail occupancy in New York as identified by a decade‐long monitoring effort (NYSDEC, unpublished data). This effort resulted in the inclusion of 14 co‐occupied sites (both New England and eastern cottontails detected at least once during study) and two sites solely occupied by New England cottontails in Putnam and Southern Dutchess counties in the lower Hudson River Valley, New York (41.5174°, −73.7191°) (Figure 2). Sites ranged in size from 0.2 to 22 ha and we delineated them as contiguous or closely associated patches of shrubland separated by a minimum of 500 m, or by linear landscape features such as roads or streams. Sites frequently comprised a mosaic of shrubland classifications, herein defined as early, mid, and late successional shrublands and persistent shrublands. Successional shrublands were characteristic of rapid shrub regeneration 5–25 years postdisturbance. Early successional shrublands were characterized by low canopy closure and had established shrubs intermixed with graminoids and tall forbs, mid‐successional shrublands had intermediate canopy closure and a dense shrub understory, and late successional shrublands were characterized by high canopy closure, low forb and grass cover, and moderate‐to‐high shrub densities. Persistent shrublands included high canopy closure ericaceous (i.e., mountain laurel, *Kalmia latifolia* and blueberry *Vaccinium* spp.) shrublands, and forested wetlands with a dense understory often consisting of sweet pepperbush (*Clethra alnifolia*) and swamp azalea (*Rhododendron viscosum*) (Appendix S1, Supporting Information). The two most abundant invasive shrubs within the study region were Japanese barberry, which has a broad range of light tolerances (Silander & Klepeis, 1999) and is common within mid and late successional shrublands, and multiflora rose, which occurred most frequently within early and mid‐successional shrublands (Appendix S1, Supporting Information). Several common native shrubs such as *Rubus* spp., dogwood (*Cornus* spp.), and *Viburnum* spp. that are palatable to New England cottontails (Pringle, 1960) were at highest densities within early successional and persistent shrublands, and typically had low densities in late successional shrublands.

Removal experiments to study competition between the cottontail species within our system were not possible given the similarity in appearance between New England and eastern cottontails, and state regulations concerning holding time of wild‐caught animals. However, sites differed in both the presence and the abundance of eastern cottontails relative to New England cottontails, permitting us to stratify resource selection analyses for New England cottontails based on the prevalence of eastern cottontails.

### 2.3 Location data acquisition

Cottontails were captured in single‐door box traps baited with apple following methods in Ryan, Gavard, Cheeseman, Cohen, and Whipps (2016). To minimize bias associated with differences in resource use, traps were placed along transects spanning entire sites at rabbit sign or every 25 m in the absence of sign. We confirmed species identity for all cottontails using two restriction digests, performed on an amplified target section of mitochondrial DNA extracted from collected ear tissue as described by Ryan et al. (2016) following modified protocols outlined by Litvaitis and Litvaitis (1996), Litvaitis, Litvaitis, Lee, and Kocher (1997), and Kovach, Litvaitis, and Litvaitis (2003). All cottontails over 800 g were affixed with a 24‐g radio collar with a zip tie closure (Advanced Telemetry Systems, Isanti, MN). Juvenile cottontails weighing less than 800 g were affixed with a 1.1‐g glue‐on radio transmitter (Advanced Telemetry Systems) with a winged‐mesh attachment following methods in Estes‐Zumpf and Rachlow (2007). Effort was made to recapture juveniles once they weighed 800 g to replace glue‐on transmitters with radio collars.

Triangulation and homing were used to locate cottontails 2–3 times weekly, year‐round (Cheeseman, 2017). All locations for each individual were obtained >24 hr apart to help ensure independence of observations. We located cottontails during both active periods (2 hr before sunset to 2 hr after sunrise) and resting periods (2 hr after sunrise to 2 hr after sunset) (Bond, Burger, Leopold, Jones, & Godwin, 2002). Homing entailed approaching each rabbit and acquiring GPS coordinates of its exact location (accuracy 5 m). Homing was the primary method employed for obtaining resting locations and was used opportunistically when obtaining active locations. Triangulation error was estimated at 27 m based on trials of transmitters placed in the field. Azimuth error was incorporated into triangulation calculations using Location of a Signal 4.0 (LOAS^TM^) software (LOAS^TM^, 2010). All work was conducted in compliance with SUNY‐ESF IACUC protocols #120801 and #151002.

### 2.4 Vegetation data acquisition

The abundance and density of shrub cover, height of cover, available herbaceous forage, and amount of tree canopy are thought to impact habitat quality for New England cottontails (Barbour & Litvaitis, 1993; Buffum et al., 2015; Warren et al., 2016); therefore, our sampling efforts sought to characterize these variables. We sampled vegetation in two seasons, defined by the availability of resources to cottontails: the leaf‐off season (November–April) and leaf‐on season (May–October). Data were collected at the centroid of every cell (hereafter “plot,” *n* = 1,191) of a 50‐m grid spanning entire sites. During the leaf‐off season, the amount of canopy closure by branches and evergreen vegetation, hereafter “persistent canopy closure,” was measured using a spherical densitometer held at a height of 1 m. To assess stem density by species, we counted the number of stems <7.5 cm dbh for all woody plant species, hereafter “shrubs,” at a height of 0.5 m within 10 × 1‐m^2^ belt transects at each plot in the leaf‐off season (Barbour & Litvaitis, 1993). During the leaf‐on season we assessed leaf‐on canopy closure, hereafter "seasonal canopy closure" as well as vegetation height and cover. Seasonal canopy closure was estimated with a spherical densitometer as above. We estimated shrub height within a gridded 1‐m^2^ quadrat at each centroid to the nearest 5 cm (Matenaar, Bazelet, & Hochkirch, 2015; Warren et al., 2016). As cover provided by forbs was seasonally variable, we separately estimated the height of forbs within the plot using the same methodology (Matenaar et al., 2015). The proportion cover of herbaceous graminoid (e.g., grasses, sedges, and rushes), shrub, and forb vegetation within each gridded 1‐m^2^ quadrat was visually estimated to the nearest 1%. To examine cottontail selection of particular vegetation types, for each plot we measured stem density of multiflora rose, Japanese barberry, and pooled palatable stems. Stem density and shrub height did not vary seasonally, so we included them in analyses for both seasons. Data were imported into ArcMap 10.4.1 (ESRI, Redlands, CA), and vegetation metrics were resampled to 10‐m resolution across sites using bilinear interpolation (Bonnot, Millspaugh, & Rumble, 2009; Stabach, Laporte, & Olupot, 2009; Vellend, Bjorkman, & McConchie, 2008).

### 2.5 Data analysis

We sampled available resources within a buffer around each used location equal in diameter to the mean movement distance of cottontail species between successive locations (Northrup, Hooten, Anderson, & Wittemyer, 2013), creating one sampling unit for each used point. We removed non‐habitat (i.e., roads, lakes, and mature forest) from the sampling unit prior to random point generation. Northrup et al. (2013) suggested that the sampling accuracy of point process models could be improved if multiple available points were paired to each used location; we therefore randomly selected up to 20 “unused” locations within each sampling unit, constrained to be far enough apart to not fall in the same pixel as other points. We subdivided used and corresponding available locations into two seasons, leaf‐on and leaf‐off, for analyses. To account for telemetry error, when assigning vegetation covariate values from our 10‐m resolution grid to used and unused points, we averaged the value from the grid cell containing the point with the values of its four neighbors (Gaston et al., 2016; Schoenecker, Nielsen, Zeigenfuss, & Pague, 2015).

To assess the effect of competition on resource selection by New England cottontails, we classified sites based on prevalence of eastern cottontails relative to New England cottontails in each year. We selected the natural break point in our data where eastern cottontails made up one in six cottontails (or 17% eastern cottontails), based on known alive animals from trapping and telemetry, which resulted in an approximately equal sample size of New England cottontail locations in both groups. Moreover, any other cutoff resulted in a sample size of New England cottontails in one category that was of negligible difference from the one in six cutoff or too small for statistical analysis, in terms of radio locations (Appendix S2, Supporting Information) and individuals (Appendix S3, Supporting Information). Hereafter, we refer to these designations as “less prevalent” where eastern cottontails made up one in six or fewer known living cottontails and “more prevalent” where eastern cottontails comprised greater than one in six known living cottontails. We also assessed resource selection by eastern cottontails where they were more prevalent than one in six cottontails, for comparison. Our sample size for eastern cottontails was not sufficient to assess their resource selection at sites where they were less prevalent. As this cutoff could be biased if a species‐specific response to trap effort existed, we assessed trapping bias using a Pearson's correlation of the ratio of Eastern to New England cottontails known to be alive from trapping and telemetry efforts to the same ratio obtained from noninvasive genetic pellet sampling for a concurrent parasitological study. Pellet survey data were available from 17 identical site and year combinations of 2014 and 2015.

We modeled resource use relative to availability separately for each species in each season as a function of seasonal tree canopy closure (leaf‐on only), persistent tree canopy closure, forb cover (leaf‐on only), shrub cover, graminoid cover (leaf‐on only), forb height (leaf‐on only), shrub height, and stem density of Japanese barberry, multiflora rose, and pooled native palatable stems (Appendix S4, Supporting Information). Stem densities were rescaled to stems/0.1 m^2^ for analysis. Models were fit using mixed‐effects conditional logistic regression in a Bayesian framework in rjags using the jags function within the jagsUI wrapper (Kellner, 2016; Plummer, 2003) in program R v. 3.2.3 (R Foundation for Statistical Computing, Vienna, Austria). We ran 3 chains in parallel for 500,000 iterations using flat normal priors for all parameters, and a burn‐in length of 100,000. We considered models converged if the R‐hat statistic was <1.1 (Gelman & Rubin, 1992). We inferred support for variables if the 95% Bayesian credible interval for the relevant regression coefficient did not overlap zero (Kéry, 2010). Because cottontails inhabit early‐ and mid‐successional stages, we expected parabolic‐shaped, intermediate selection of most vegetation characteristics. As a result, we initially incorporated quadratic effects for all variables except forb height, then removed quadratic effects where the credible interval overlapped zero from the models. To account for repeated measures and autocorrelation among locations, we used individual as a random effect for all coefficients (Duchesne, Fortin, & Courbin, 2010; Gillies et al., 2006; McCall et al., 2016). As selection may be influenced by variation among sites and years, we considered hierarchical models of individual nested within year or site. In a mixed conditional logistic regression model, each regression coefficient (*β*_i_) has an associated standard deviation among levels of the random effect (*σ*_i_) which represents the variation in resource use due to the random effect. Moreover, each *β*_i_ and *σ*_i_ is estimated with sampling error (SE*^β^*_i_ and SE*^σ^*_i_) which in a Bayesian analysis are the standard deviations of the posterior distributions. If, for a particular random effect, *σ*_i_ is high relative to *β*_i,_ and if SE^σ^_i_ is low relative to *σ*_i_, then there is evidence for the importance of including that random effect in the model (McCall et al., 2016), so we used these ratios as criteria for keeping particular random effects in our model. Comparisons between seasons, species, and eastern cottontail prevalence categories were made by examining the degree of overlap in prediction intervals for each variable. We interpreted selection or avoidance for a range of values where prediction intervals did not overlap 0.5 and a difference in selection or avoidance of particular resources where the prediction intervals overlapped by less than 25% (Cumming & Finch, 2005).

### 2.6 Mapping predictions

We mapped predicted resource use for each value relative to the mean shrubland condition to facilitate comparison across sites. We extracted site‐specific resource values for each 10‐m raster cell and calculated predicted use and 95% prediction intervals for each cell using study‐level average resource values as the available reference for each plot. Predicted resource use and 95% prediction intervals were then mapped in ArcMap 10.4.1.

## 3 RESULTS

We collared 80 New England cottontails and 68 eastern cottontails and collected a total of 5,375 locations to be used in resource selection analyses (Table 1). The number of New England cottontails monitored per site ranged from 1 to 11 (x̅ = 5.2) and the number of eastern cottontails ranged from 1 to 14 (x̅ = 5.7; Appendix S5, Supporting Information). Available resources did not differ between eastern cottontail “more prevalent” and “less prevalent” sites in any consistent manner among categories of shrubland (Appendix S6, Supporting Information). The proportion of eastern cottontails within five sites changed between years; we accordingly changed their designation for our analyses (Table 1). We detected no species‐specific bias in trapping, based on correlation between species composition in our captured population and in pellet samples (*r* = 0.642, *n* = 17 site x year combinations, *p* = 0.005). Mean movement distance between successive locations did not differ between cottontail species and equaled 75 m (Cheeseman, 2017). As such, we defined available locations as those within 75 m of each used coordinate. To create vegetation data layers, we sampled vegetation in 1,191 plots in both leaf‐off and leaf‐on seasons.

**Table 1:** Sample sizes for cottontails at 16 sites used in resource selection models

Species	Prevalence	Sites	Leaf‐on season[Link]	Leaf‐off season[Link]
New England cottontail	More prevalent	11[Link]	707 (16)	979 (35)
Eastern cottontail	More prevalent	11[Link]	934 (26)	1,196 (38)
New England cottontail	Less prevalent	10	704 (15)	855 (24)

Note

Data are shown as number of locations (number of individuals), of New England and eastern cottontails by season and eastern cottontail prevalence categories.

May through October.

November through April.

Prevalance category was assessed annually and the assigned prevalence category changed for five sites during the course of the study.

Sites used in assessing New England and eastern cottontail resource use where eastern cottontails were more prevalent were identical.

For all models, σ_i_ was high relative to β_i_ and all SE^σ^_I_ were low relative to σ_i_ for the random effect of individual in our models for New England cottontails (Appendix 7, Supporting Information) and eastern cottontails (Appendix 8, Supporting Information). However, all SE^σ^_i_ were high relative to σ_i_ for the random effects of site and year, such that we had little evidence for their importance as a source of variation in resource use. Thus, we proceeded to make inferences with models including only the random effect of individual.

### 3.1 Resource selection

**Figure 3:**
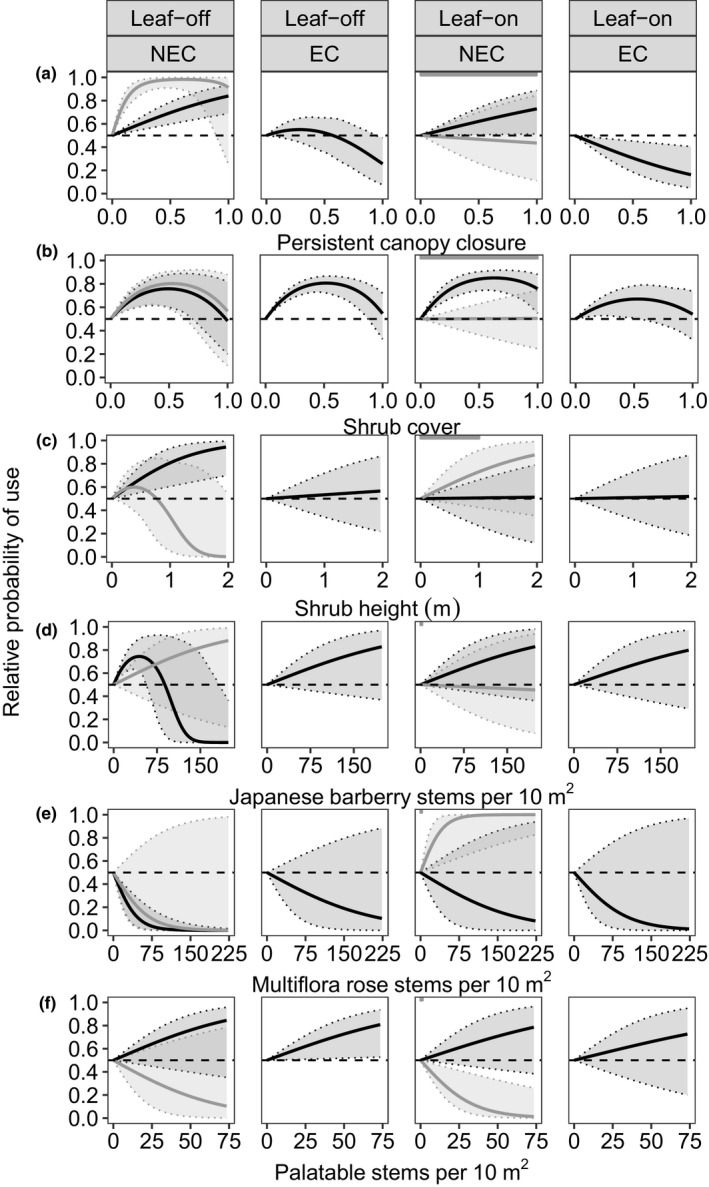
Relative probability of resource use versus vegetation characteristics for New England cottontails (NEC, *Sylvilagus transitionalis*) and eastern cottontails (EC, *S. floridanus)* in the leaf‐off season (columns 1 and 2) and leaf‐on season (columns 3 and 4) in New York, 2013–2016. For NEC (columns 1 and 3), the gray line depicts the predicted relationship for sites where EC were less prevalent and the black line depicts the relationship for sites where EC were more prevalent. For EC (column 2 and 4), predictions are only shown for sites where EC were more prevalent. The horizontal dashed line indicates probability of use equal to 0.5 (no selection). Evidence for importance of a range of values for each variable is inferred where 95% prediction intervals (shaded areas) do not overlap 0.5.

New England cottontail resource selection varied seasonally and between categories of eastern cottontail prevalence. For example, New England cottontails displayed intermediate selection for low‐to‐moderate values of persistent canopy closure (Figure 3a) and shrub cover (Figure 3b) during the leaf‐off season where eastern cottontails were less prevalent. At sites where eastern cottontails were more prevalent and in the leaf‐off season, probability of use by New England cottontails gradually increased with persistent canopy closure (Figure 3a) and shrub height (Figure 3c), with the highest use at high values of canopy closure. In these areas, New England cottontails also selected for low‐to‐moderate values of Japanese barberry with use highest at values associated with mid‐ and late successional shrublands (50 stems per 10 m^2^; Figure 3d). New England cottontails avoided high densities of Japanese barberry and shade‐intolerant multiflora rose where eastern cottontails were more prevalent in the leaf‐off season (Figure 3e). As where eastern cottontails were less prevalent, New England cottontails also selected for moderate values of proportion shrub cover in these areas (Figure 3b).

**Figure 4:**
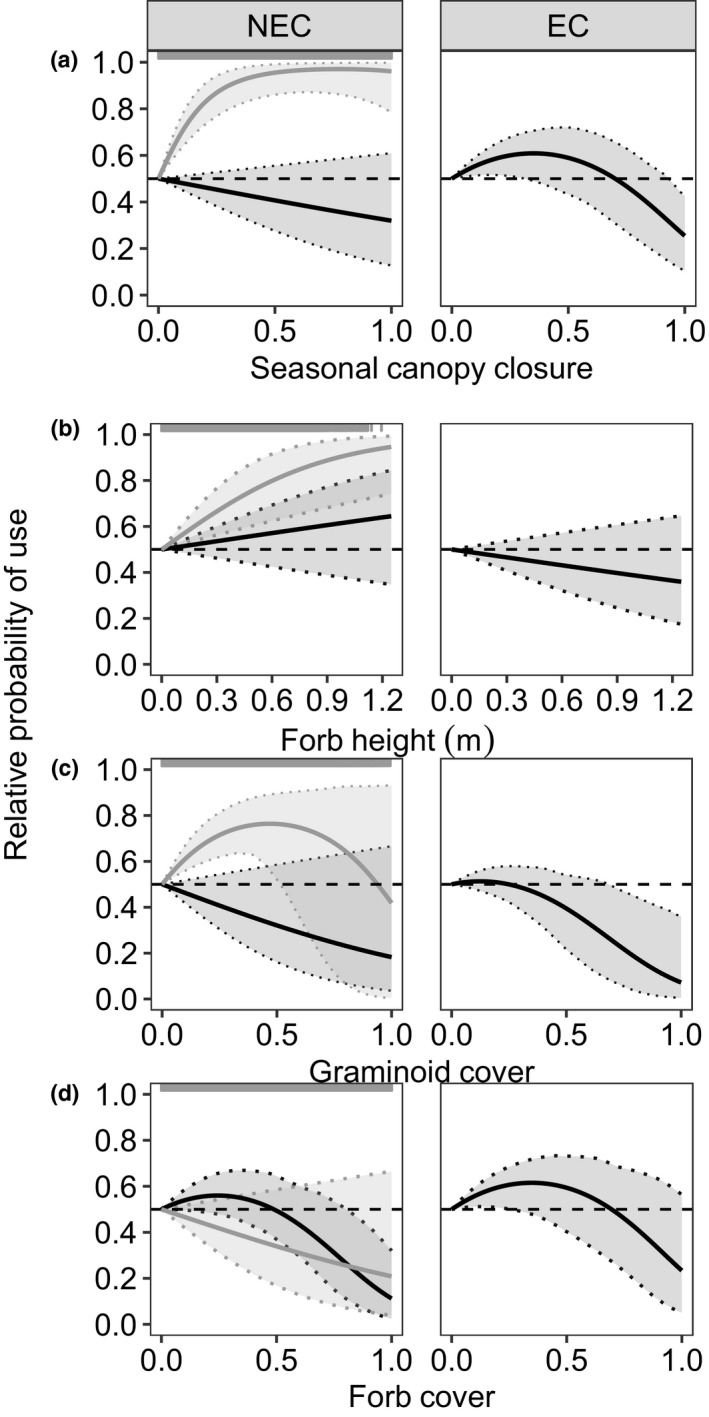
Relative probability of resource use versus leaf‐on vegetation characteristics for New England cottontails (NEC, *Sylvilagus transitionalis*) and eastern cottontails (EC, *S. floridanus*). For NEC (column 1), the gray line depicts the predicted relationship for sites where EC were less prevalent and the black line depicts the relationship for sites where EC were more prevalent. For EC (column 2), predictions are only shown for sites where EC were more prevalent. The horizontal dashed line indicates probability of use equal to 0.5 (no selection). Evidence for importance of a range of values for each variable is inferred where 95% prediction intervals (shaded areas) do not overlap 0.5.

Where eastern cottontails were less prevalent in the leaf‐on season, New England cottontails did not select for shrub cover, but did use densities of multiflora rose characteristic of early‐ and mid‐successional shrublands (Figure 3e), and avoided native palatable stems (Figure 3f). In these areas, New England cottontails displayed strong selection of seasonal canopy starting at low proportion closure (Figure 4a), selected for tall forbs (Figure 4b), and low‐to‐intermediate proportion graminoid cover (Figure 4c), all characteristic of early successional shrublands. However, where eastern cottontails were more prevalent in the leaf‐on season, New England cottontails selected for the high values of persistent canopy closure (Figure 3a) typified by mid‐ and late successional forests, and moderate‐to‐high values of shrub cover (Figure 3b).

In the leaf‐off season, eastern cottontails showed slight use of areas with little or no persistent canopy closure and avoidance of high canopy closure (Figure 3a). They also selected for moderate shrub cover (Figure 3b) and native palatable stems (Figure 3f) during the leaf‐off season. During the leaf‐on season, eastern cottontails avoided persistent canopy closure (Figure 3b), and they selected for low shrub cover (Figure 3c) although not as strongly as during the leaf‐off season. Eastern cottontails also selected for low seasonal canopy closure (Figure 4a) and forb cover (Figure 4d), but avoided high graminoid cover in the leaf‐on season (Figure 4c).

**Figure 5:**
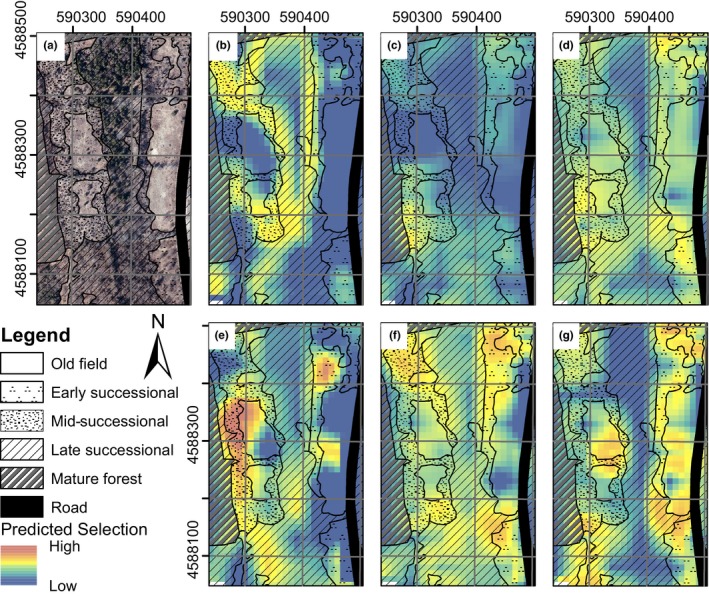
Example of (a) site‐level cover types and (b) predicted resource selection in the leaf‐off season by New England cottontails where eastern cottontails were less prevalent, (c) New England cottontails where eastern cottontails were more prevalent, (d) eastern cottontails where they were more prevalent, and (e) predicted resource selection in the leaf‐on season by New England cottontails where eastern cottontails were less prevalent, (f) New England cottontails where eastern cottontails were more prevalent and (g) eastern cottontails where they were more prevalent. Predicted selection calculated for each pixel when available resources were held at shrubland means.

When resource use was predicted at the site level, integrating all model variables during the leaf‐off season, and where eastern cottontails were less prevalent, New England cottontails selected for resources associated with mid‐successional shrubland and forest edges (Figure 5, 95% prediction intervals displayed in Appendices S9 and S10, Supporting Information). However, where eastern cottontails were more prevalent in the leaf‐off season, New England cottontail resource selection was more characteristic of interior late successional shrublands than mid‐successional shrublands and forest edges (Figure 5). During the leaf‐on season, where eastern cottontails were less prevalent, New England cottontail resource selection was characteristic of early‐ to mid‐successional shrublands (Figure 5). In contrast, where eastern cottontails were more prevalent, New England cottontail relative probability of use during the leaf‐on season was characterized by resources consistent with mid‐ to late successional shrublands (Figure 5). Shifts in resource use by New England cottontails, in sites where the eastern cottontail prevalence designation shifted between years, were also evident (Appendix 11, Supporting Information). Eastern cottontail use was characteristic of early successional shrublands in both seasons, with only slight shifts in use toward resources characteristic of older successional shrublands during the leaf‐off season (Figure 5, 95% prediction intervals reported in Appendices S8 and S9, Supporting Information).

## 4 DISCUSSION

We provide evidence for displacement of a native species by an introduced species from otherwise selected resources. Moreover, the use of low‐to‐moderate densities of the common invasive shrub Japanese barberry was higher where the introduced competitor was more prevalent than where it was less prevalent. Competitive displacement by nonnative species has resulted in negative demographic effects and has led to population declines and extinctions (reviewed in Mooney & Cleland, 2001 and Harris, 2009). For example, competition with gray squirrels *Sciurus carolinensis* over food resources has led to widespread declines in Eurasian red squirrel *S. vulgaris* where the two are now sympatric (Bertolino, Montezemolo, Preatoni, Wauters, & Martinoli, 2014). In this study, we observed evidence that New England cottontails were displaced into later successional‐stage shrublands where eastern cottontails are prevalent. Of particular concern is displacement of New England cottontails into Japanese barberry and tall shrubs in the leaf‐off season where eastern cottontails were more prevalent. Although tall shrubs and Japanese barberry may provide additional and novel escape cover, habitat quality and forage availability influence overwinter survival of New England cottontails (Smith & Litvaitis, 2000; Villafuerte et al., 1997) and these resources may not provide adequate forage (Warren et al., 2016). Furthermore, high tick burdens have been linked to population crashes in cottontails (Smith & Cheatum, 1944), and for New England cottontails, higher tick burdens have been observed where sites are dominated by invasive vegetation, including Japanese barberry (Mello, 2018). Given the potential for competitive displacement of New England cottontails from early‐ to mid‐successional shrublands where eastern cottontails are present, traditional methods of successional shrubland management (i.e., clearcutting, controlled burns, and brush‐hogging) that restore forests to grassland or early successional shrubland over large areas will benefit nonnative eastern cottontails over New England cottontails. As such, it may be difficult for managers to meet New England cottontail restoration goals using traditional shrubland management practices where eastern cottontails are present. Under these conditions, management of eastern cottontails or selective habitat management may be necessary.

Eradication of nonnative mammals over large areas has proven challanging, and successful eradications can have undesired consequences, resulting in degraded ecosystems or hyperpredation of native fauna (Aguirre‐Muñoz et al., 2008; Lees & Bell, 2008; Zavaleta et al., 2001). Where competition with a nonnative species is a conservation concern and eradication is undesirable or infeasible, managing habitat to improve survival or recruitment of native species is an option (Cole et al., 2005). In European forests, management to promote hazel *Corylus avellana* over oak *Quercus robur* within deciduous forests has been recommended to alter competitive interactions in favor of the native Eurasian red squirrel where it is being displaced by invasive gray squirrels (Kenward & Holm, 1993). Here, we present evidence that New England cottontails use shrublands with high canopy closure but that eastern cottontails avoid these areas. This is similar to the competitive interactions hypothesized to occur between Appalachian and eastern cottontails, where Appalachian cottontails are displaced from successional shrublands into mature forest and ericaceous shrublands (Russell et al., 1999). We suggest that where competition with eastern cottontails is a concern and eastern cottontail removal is deemed infeasible, managing for large patches of dense, high canopy closure successional shrublands, ericaceous shrublands with intermixed gap phase processes, or shrub‐covered forested wetlands would allow for New England cottontails use, without encouraging use by eastern cottontails. Adaptive management of habitat based on our hypotheses and analysis of the fitness consequences of resource selection by New England cottontails in our system are ongoing.

Antagonistic interactions between resident New England cottontails and colonizing eastern cottontails that could explain altered resource use by New England cottontails in the presence of competing eastern cottontails have not been observed (Probert & Litvaitis, 1996). However, the ability for generalist eastern cottontails to colonize early successional shrublands before they are suitable for New England cottontail occupancy may limit colonization by New England cottontails (Probert & Litvaitis, 1996) and could partially explain the pattern of resource use observed here. It is also probable that a similar form of scramble competition occurs within sites at the scale of resource selection, where eastern cottontails use areas at an earlier successional stage than is suitable for New England cottontails, and are able to hold them as they mature into suitability. Moreover, New England cottontail resource selection shifted seasonally regardless of eastern cottontail prevalence, from dense, high canopy shrublands in the leaf‐off season to less dense, open‐canopy shrublands in the leaf‐on season, likely in response to seasonal availability of forage and apparent cover provided by tall forbs in the leaf‐on season. We propose that seasonal shifts away from resources associated with early successional shrublands result in a small‐scale form of competitive release in these areas benefiting eastern cottontails. Individual niche expansion resulting from competitive release is documented (Bodey, McDonald, & Bearhop, 2009; Bolnick et al., 2010), and such behavioral responses by resident eastern cottontails to seasonal competitive release within early successional shrublands provide a mechanism for competitive displacement of New England cottontails that is not reliant on patterns of colonization or displacement through antagonistic interactions.

## 5 CONCLUSIONS

Using conditional resource selection functions, we observed that resource selection, including selection for common invasive plants, by the imperiled native New England cottontail varied by the relative prevalence of a nonnative competitor. Our results suggest seasonal competitive release as a mechanism that facilitates competitive displacement of resident New England cottontails by its nonnative competitor. We caution that current management strategies may benefit the nonnative competitor over the imperiled target species. Our results suggest silvicultural approaches such as clearcutting or mowing to create successional shrublands may favor the nonnative competitor. Instead, we suggest seed tree cuts, shelter wood cuts, or selective thinning to create canopy gaps could be used to adaptively manage sites to promote the native species where its nonnative competitor is present. Treatments should vary by site and consider present context, the presence of shade‐tolerant invasive shrubs, and anticipated shrubland density under different treatment scenarios, selecting a treatment that balances high canopy closure with dense native understory regeneration. Where native shrubs are not present, seeding or planting of native shrubs and regular management to minimize invasive shrub recruitment may be required to encourage native shrub regeneration. Our approach can be broadly applied to other situations where fine‐scale resource use data are available, and can inform invasive species management decisions and habitat management in the presence of competing species within the ecosystem context and at a scale relevant to site‐level habitat management.

## AUTHORS’ CONTRIBUTIONS

AEC and JBC conceived of the ideas and designed methodology with input from CMW and SJR. AEC collected the data. Genetic work was supervised by CMW. AEC and JBC analyzed the data. AEC and JBC led manuscript preparation. All authors contributed substantially to drafts and gave final approval for publication.

## DATA ACCESSIBILITY

Data available from the Dryad Digital Repository: https://doi.org/10.5061/dryad.72qb1k2.

## References

[ece34580-bib-0001] Aguirre‐Muñoz, A. , Croll, D. A. , Donlan, C. J. , Henry, R. W. III , Hermosillo, M. A. , Howald , … L. M. (2008). High‐impact conservation: Invasive mammal eradications from the islands of western Mexico. AMBIO: A Journal of the Human Environment, 37, 101–107. 10.1579/0044-7447(2008)37[101:hcimef]2.0.co;2 18488552

[ece34580-bib-0002] Ballari, S. A. , Kuebbing, S. E. , & Nuñez, M. A. (2016). Potential problems of removing one invasive species at a time: A meta‐analysis of the interactions between invasive vertebrates and unexpected effects of removal programs. PeerJ, 4, e2029. 27280066 10.7717/peerj.2029 PMC4893336

[ece34580-bib-0003] Barbour, M. S. , & Litvaitis, J. A. (1993). Niche dimensions of New England cottontails in relation to habitat patch size. Oecologia, 95, 321–327. 10.1007/BF00320983 28314005

[ece34580-bib-0004] Bertolino, S. , Di Montezemolo, N. C. , Preatoni, D. G. , Wauters, L. A. , & Martinoli, A. (2014). A grey future for Europe: Sciurus carolinensis is replacing native red squirrels in Italy. Biological Invasions, 16, 53–62. 10.1007/s10530-013-0502-3

[ece34580-bib-0005] Bodey, T. W. , McDonald, R. A. , & Bearhop, S. (2009). Mesopredators constrain a top predator: Competitive release of ravens after culling crows. Biology Letters, 5, 617–620. 10.1098/rsbl.2009.0373 19570777 PMC2781970

[ece34580-bib-0006] Bolnick, D. I. , Ingram, T. , Stutz, W. E. , Snowberg, L. K. , Lau, O. L. , & Paull, J. S. (2010). Ecological release from interspecific competition leads to decoupled changes in population and individual niche width. Proceedings of the Royal Society of London B: Biological Sciences, 277, 1789–1797. 10.1098/rspb.2010.0018 PMC2871882 20164100

[ece34580-bib-0007] Bond, B. T. , Burger, L. W. , Leopold, B. D. , Jones, J. C. , & Godwin, K. D. (2002). Habitat use by cottontail rabbits across multiple spatial scales in Mississippi. Journal of Wildlife Management, 66, 1171–1178. 10.2307/3802950

[ece34580-bib-0008] Bonnot, T. W. , Millspaugh, J. J. , & Rumble, M. A. (2009). Multi‐scale nest‐site selection by black‐backed woodpeckers in outbreaks of mountain pine beetles. Forest Ecology and Management, 259, 220–228. 10.1016/j.foreco.2009.10.021

[ece34580-bib-0009] Buffum, B. , McGreevy, T. J. Jr , Gottfried, A. E. , Sullivan, M. E. , & Husband, T. P. (2015). An analysis of overstory tree canopy cover in sites occupied by native and introduced cottontails in the Northeastern United States with recommendations for habitat management for New England cottontail. PLoS One, 10, e0135067. 26267857 10.1371/journal.pone.0135067 PMC4534376

[ece34580-bib-0010] Cheeseman, A. E. (2017). Factors limiting the recovery of the New England cotttontail in New York. Syracuse, NY: Dissertation, State University of New York College of Environmental Science and Forestry.

[ece34580-bib-0011] Cole, N. C. , Jones, C. G. , & Harris, S. (2005). The need for enemy‐free space: The impact of an invasive gecko on island endemics. Biological Conservation, 125, 467–474. 10.1016/j.biocon.2005.04.017

[ece34580-bib-0012] Cumming, G. , & Finch, S. (2005). Inference by eye: Confidence intervals and how to read pictures of data. American Psychologist, 60, 170–180. 10.1037/0003-066X.60.2.170 15740449

[ece34580-bib-0013] Dalke, P. D. , & Sime, P. R. (1941). Food habits of the eastern and New England cottontails. Journal of Wildlife Management, 5, 216–228. 10.2307/3795589

[ece34580-bib-0014] DeGrandchamp, K. L. , Garvey, J. E. , & Colombo, R. E. (2008). Movement and habitat selection by invasive Asian carps in a large river. Transactions of the American Fisheries Society, 137, 45–56. 10.1577/T06-116.1

[ece34580-bib-0015] Douglas, M. E. , Marsh, P. C. , & Minckley, W. (1994). Indigenous fishes of western North America and the hypothesis of competitive displacement: *Meda fulgida* (Cyprinidae) as a case study. Copeia, 9–19. 10.2307/1446665

[ece34580-bib-0016] Duchesne, T. , Fortin, D. , & Courbin, N. (2010). Mixed conditional logistic regression for habitat selection studies. Journal of Animal Ecology, 79, 548–555. 10.1111/j.1365-2656.2010.01670.x 20202010

[ece34580-bib-0017] Estes‐Zumpf, W. A. , & Rachlow, J. L. (2007). Evaluation of radio‐transmitters on juvenile rabbits: Application to the semifossorial pygmy rabbit (*Brachylagus idahoensis*). Western North American Naturalist, 67, 133–136. 10.3398/1527-0904(2007)67[133:EOROJR]2.0.CO;2

[ece34580-bib-0018] Fenderson, L. E. , Kovach, A. I. , Litvaitis, J. A. , & Litvaitis, M. K. (2011). Population genetic structure and history of fragmented remnant populations of the New England cottontail (*Sylvilagus transitionalis*). Conservation Genetics, 12, 943–958. 10.1007/s10592-011-0197-x

[ece34580-bib-0019] Foster, D. R. , Motzkin, G. , Bernardos, D. , & Cardoza, J. (2002). Wildlife dynamics in the changing New England landscape. Journal of Biogeography, 29, 1337–1357. 10.1046/j.1365-2699.2002.00759.x

[ece34580-bib-0020] Fuller, S. , & Tur, A. (2012) Conservation strategy for the New England cottontail (Sylvilagus transitionalis). pp. 143 Retrieved from https://newenglandcottontail.org/resource/conservation-strategy-new-england-%20cottontail

[ece34580-bib-0021] Gaston, A. , Blazquez‐Cabrera, S. , Garrote, G. , Mateo‐Sanchez, M. C. , Beier, P. , Simon, M. A. , & Saura, S. (2016). Response to agriculture by a woodland species depends on cover type and behavioural state: Insights from resident and dispersing Iberian lynx. Journal of Applied Ecology, 53, 814–824.

[ece34580-bib-0022] Gelman, A. , & Rubin, D. B. (1992). Inference from iterative simulation using multiple sequences. Statistical Science, 7, 457–472. 10.1214/ss/1177011136

[ece34580-bib-0023] Gillies, C. S. , Hebblewhite, M. , Nielsen, S. e. , Krawchuk, M. A. , Aldridge, C. L. , Frair, J. L. , … Jerde, C. L. (2006). Application of random effects to the study of resource selection by animals. Journal of Animal Ecology, 75, 887–898. 10.1111/j.1365-2656.2006.01106.x 17009752

[ece34580-bib-0024] Glen, A. S. , Atkinson, R. , Campbell, K. J. , Hagen, E. , Holmes, N. D. , Keitt, B. S. , … Torres, H. (2013). Eradicating multiple invasive species on inhabited islands: The next big step in island restoration? Biological Invasions, 15, 2589–2603. 10.1007/s10530-013-0495-y

[ece34580-bib-0025] Gurevitch, J. , & Padilla, D. K. (2004). Are invasive species a major cause of extinctions? Trends in Ecology and Evolution, 19, 470–474. 10.1016/j.tree.2004.07.005 16701309

[ece34580-bib-0026] Harris, D. B. (2009). Review of negative effects of introduced rodents on small mammals on islands. Biological Invasions, 11, 1611–1630. 10.1007/s10530-008-9393-0

[ece34580-bib-0027] Johnson, V. S. , Litvaitis, J. A. , Lee, T. D. , & Frey, S. D. (2006). The role of spatial and temporal scale in colonization and spread of invasive shrubs in early successional habitats. Forest Ecology and Management, 228, 124–134. 10.1016/j.foreco.2006.02.033

[ece34580-bib-0028] Kellner, K. (2016). A Wrapper Around 'rjags' to Streamline 'JAGS' Analyses. R package version 1.4.4. Retrieved from https://CRAN.R-project.org/package=jagsUI.

[ece34580-bib-0029] Kenward, R. , & Holm, J. (1993) On the replacement of the red squirrel in Britain: a phytotoxic explanation. Proceedings of the Royal Society B, 251, 187–194. 10.1098/rspb.1993.0028 8097326

[ece34580-bib-0030] Kéry, M. (2010). Introduction to WinBUGS for ecologists. Burlington, MA: Academic Press.

[ece34580-bib-0031] Kovach, A. I. , Litvaitis, M. K. , & Litvaitis, J. A. (2003). Evaluation of fecal mtDNA analysis as a method to determine the geographic distribution of a rare lagomorph. Wildlife Society Bulletin, 31, 1061–1065.

[ece34580-bib-0032] Lees, A. C. , & Bell, D. J. (2008). A conservation paradox for the 21st century: The European wild rabbit *Oryctolagus cuniculus*, an invasive alien and an endangered native species. Mammal Review, 38, 304–320.

[ece34580-bib-0033] Litvaitis, J. A. (1993). Response of early successional vertebrates to historic changes in land‐use. Conservation Biology, 7, 866–873. 10.1046/j.1523-1739.1993.740866.x

[ece34580-bib-0034] Litvaitis, J. A. , Barbour, M. S. , Brown, A. L. , Kovach, A. I. , Litvaitis, M. K. , Oehler, J. D. , … Villafuerte, R. (2008). Testing multiple hypotheses to identify causes of the decline of a lagomorph species: The New England cottontail as a case study In Alves P. C., Ferrand N., & Hacklander K. (Eds.), Lagomorph Biology: Evolution, Ecology, and Conservation (pp. 167–185). Berlin, Germany: Springer‐Verlag.

[ece34580-bib-0035] Litvaitis, M. K. , & Litvaitis, J. A. (1996). Using mitochondrial DNA to inventory the distribution of remnant populations of New England cottontails. Wildlife Society Bulletin, 24, 725–730.

[ece34580-bib-0036] Litvaitis, M. K. , Litvaitis, J. A. , Lee, W. J. , & Kocher, T. D. (1997). Variation in the mitochondrial DNA of the *Sylvilagus* complex occupying the northeastern United States. Canadian Journal of Zoology, 75, 595–605. 10.1139/z97-074

[ece34580-bib-0037] Litvaitis, J. A. , Tash, J. P. , Litvaitis, M. K. , Marchand, M. N. , Kovach, A. I. , & Innes, R. (2006). A range‐wide survey to determine the current distribution of New England cottontails. Wildlife Society Bulletin, 34, 1190–1197. 10.2193/0091-7648(2006)34[1190:ARSTDT]2.0.CO;2

[ece34580-bib-0038] Matenaar, D. , Bazelet, C. S. , & Hochkirch, A. (2015). Simple tools for the evaluation of protected areas for the conservation of grasshoppers. Biological Conservation, 192, 192–199. 10.1016/j.biocon.2015.09.023

[ece34580-bib-0039] McCall, A. G. , Pilfold, N. W. , Derocher, A. E. , & Lunn, N. J. (2016). Seasonal habitat selection by adult female polar bears in western Hudson Bay. Population Ecology, 58, 407–419. 10.1007/s10144-016-0549-y

[ece34580-bib-0040] Mello, S. L. (2018). Parasites of the New England cottontail (*Sylvilagus transitionalis*) in the presence of a non‐native hose and invasive vegetation, M.S. Thesis Syracuse, NY: State University of New York College of Environmental Science and Forestry.

[ece34580-bib-0041] Mooney, H. A. , & Cleland, E. E. (2001). The evolutionary impact of invasive species. Proceedings of the National Academy of Sciences, 98, 5446–5451. 10.1073/pnas.091093398 PMC33232 11344292

[ece34580-bib-0042] Morgan, K. A. , & Gates, J. E. (1983). Use of forest edge and strip vegetation by eastern cottontails. Journal of Wildlife Management, 47, 259–264. 10.2307/3808081

[ece34580-bib-0043] Nelson, E. W. (1909). Rabbits of North America vol 29. Washingtion DC: North American Fauna United States Department of Agriculture.

[ece34580-bib-0044] Northrup, J. M. , Hooten, M. B. , Anderson, C. R. , & Wittemyer, G. (2013). Practical guidance on characterizing availability in resource selection functions under a use–availability design. Ecology, 94, 1456–1463. 10.1890/12-1688.1 23951705

[ece34580-bib-0045] Plummer, M. (2003). JAGS: A program for analysis of Bayesian graphical models using Gibbs sampling In Hornik K., Leisch F., & Zeileis A. (Eds.), Proceedings of the 3rd international workshop on distributed statistical computing (pp. 125). Vienna, Austria:DSC.

[ece34580-bib-0046] Pressey, R. L. , Cabeza, M. , Watts, M. E. , Cowling, R. M. , & Wilson, K. A. (2007). Conservation planning in a changing world. Trends in Ecology & Evolution, 22, 583–592. 10.1016/j.tree.2007.10.001 17981360

[ece34580-bib-0047] Pringle, L. P. (1960). A study of the biology and ecology of the New England cottontail (Sylvilagus transitionalis) in Massachusetts. M.S. thesis. Amherst, MA: University of Massachusetts.

[ece34580-bib-0048] Probert, B. L. , & Litvaitis, J. A. (1996). Behavioral interactions between invading and endemic lagomorphs: Implications for conserving a declining species. Biological Conservation, 76, 289–295. 10.1016/0006-3207(95)00127-1

[ece34580-bib-0049] Recio, M. , Mathieu, R. , Virgós, E. , & Seddon, P. (2014). Quantifying fine‐scale resource selection by introduced feral cats to complement management decision‐making in ecologically sensitive areas. Biological Invasions, 16, 1915–1927. 10.1007/s10530-013-0635-4

[ece34580-bib-0050] Rodriguez, L. F. (2006). Can invasive species facilitate native species? Evidence of how, when, and why these impacts occur. Biological Invasions, 8, 927–939. 10.1007/s10530-005-5103-3

[ece34580-bib-0051] Russell, K. R. , Moorman, C. E. , & Guynn, J. R. D. C. (1999). Appalachian cottontails, *Sylvilagus obscurus* (Lagomorpha: Leporidae), from the South Carolina mountains with observations on habitat use. Journal of the Elisha Mitchell Scientific Society, 115, 140–144.

[ece34580-bib-0052] Russo, D. , Jones, G. , & Migliozzi, A. (2002). Habitat selection by the Mediterranean horseshoe bat, *Rhinolophus euryale* (Chiroptera: Rhinolophidae) in a rural area of southern Italy and implications for conservation. Biological Conservation, 107, 71–81. 10.1016/S0006-3207(02)00047-2

[ece34580-bib-0053] Ryan, S. J. , Gavard, E. J. , Cheeseman, A. E. , Cohen, J. B. , & Whipps, C. M. (2016). Reference and baseline hematocrit measures for the threatened New England cottontail (*Sylvilagus transitionalis*) and comparison with sympatic eastern cottontail (*Sylvilagus floridanus*) rabbits. Journal of Zoo and Wildlife Medicine, 47, 659–662. 10.1638/2015-0157.1 27468046

[ece34580-bib-0054] Schoenecker, K. A. , Nielsen, S. E. , Zeigenfuss, L. C. , & Pague, C. A. (2015). Selection of vegetation types and density of bison in an arid ecosystem. Journal of Wildlife Management, 79, 1117–1128. 10.1002/jwmg.940

[ece34580-bib-0055] Schroeder, N. , Ovejero, R. , Moreno, P. G. , Gregorio, P. , Taraborelli, P. , Matteucci, S. D. , & Carmanchahi, P. D. (2013). Including species interactions in resource selection of guanacos and livestock in Northern Patagonia. Journal of Zoology, 291, 213–225. 10.1111/jzo.12065

[ece34580-bib-0056] Silander, J. A. , & Klepeis, D. M. (1999). The invasion ecology of Japanese barberry (*Berberis thunbergii*) in the New England landscape. Biological Invasions, 1, 189–201.

[ece34580-bib-0057] Smith, R. H. , & Cheatum, E. L. (1944). Role of ticks in decline of an insular cottontail population. The Journal of Wildlife Management, 8, 311–317. 10.2307/3796026

[ece34580-bib-0058] Smith, D. F. , & Litvaitis, J. A. (2000). Foraging strategies of sympatric lagomorphs: Implications for differential success in fragmented landscapes. Canadian Journal of Zoology, 78, 2134–2141. 10.1139/z00-160

[ece34580-bib-0059] Stabach, J. A. , Laporte, N. , & Olupot, W. (2009). Modeling habitat suitability for Grey Crowned‐cranes (*Balearica regulorum gibbericeps*) throughout Uganda. International Journal of Biodiversity and Conservation, 1, 177–186.

[ece34580-bib-0060] Stewart, K. M. , Bowyer, R. T. , Kie, J. G. , Cimon, N. J. , & Johnson, B. K. (2002). Temporospatial distributions of elk, mule deer, and cattle: Resource partitioning and competitive displacement. Journal of Mammalogy, 83, 229–244. 10.1644/1545-1542(2002)083<0229:TDOEMD>2.0.CO;2

[ece34580-bib-0061] Sweetman, H. L. (1949). Further studies of the winter feeding habits of cottontail rabbits. Ecology, 30, 371–376. 10.2307/1932618

[ece34580-bib-0062] Thomas, D. L. , & Taylor, E. J. (2006). Study designs and tests for comparing resource use and availability II. Journal of Wildlife Management, 70, 324–336. 10.2193/0022-541X(2006)70[324:SDATFC]2.0.CO;2

[ece34580-bib-0063] Van Riel, P. , Jordaens, K. , Martins, A. M. F. , & Backeljau, T. (2000). Eradication of exotic species. Trends in Ecology & Evolution, 15, 515 10.1016/S0169-5347(00)02007-3

[ece34580-bib-0064] Vellend, M. , Bjorkman, A. D. , & McConchie, A. (2008). Environmentally biased fragmentation of oak savanna habitat on southeastern Vancouver Island, Canada. Biological Conservation, 141, 2576–2584. 10.1016/j.biocon.2008.07.019

[ece34580-bib-0065] Villafuerte, R. , Litvaitis, J. A. , & Smith, D. F. (1997). Physiological responses by lagomorphs to resource limitations imposed by habitat fragmentation: Implications for condition‐sensitive predation. Canadian Journal of Zoology, 75, 148–151. 10.1139/z97-019

[ece34580-bib-0066] Warren, A. , Litvaitis, J. A. , & Keirstead, D. (2016). Developing a habitat suitability index to guide restoration of New England cottontail habitats. Wildlife Society Bulletin, 40, 69–77. 10.1002/wsb.616

[ece34580-bib-0067] Wauters, L. A. , Gurnell, J. , Martinoli, A. , & Tosi, G. (2002). Interspecific competition between native Eurasian red squirrels and alien grey squirrels: Does resource partitioning occur? Behavioral Ecology and Sociobiology, 52, 332–341. 10.1007/s00265-002-0516-9

[ece34580-bib-0068] Westhoff, J. T. , & Rabeni, C. F. (2013). Resource selection and space use of a native and an invasive crayfish: Evidence for competitive exclusion? Freshwater Science, 32, 1383–1397. 10.1899/13-036.1

[ece34580-bib-0069] Wilcove, D. S. , Rothstein, D. , Dubow, J. , Phillips, A. , & Losos, E. (1998). Quantifying threats to imperiled species in the United States. BioScience, 48, 607–615. 10.2307/1313420

[ece34580-bib-0070] Yurkonis, K. A. , Meiners, S. J. , & Wachholder, B. E. (2005). Invasion impacts diversity through altered community dynamics. Journal of Ecology, 93, 1053–1061. 10.1111/j.1365-2745.2005.01029.x

[ece34580-bib-0071] Zavaleta, E. S. , Hobbs, R. J. , & Mooney, H. A. (2001). Viewing invasive species removal in a whole‐ecosystem context. Trends in Ecology & Evolution, 16, 454–459. 10.1016/S0169-5347(01)02194-2

