# Supplementary material for: Competition alters seasonal resource selection and promotes use of invasive shrubs by an imperiled native cottontail
Source: Ecol Evol. 2018 Oct 25;8(22):11122–33. doi: 10.1002/ece3.4580 (PMC6262724; doi:10.1002/ece3.4580)
Supplement: Supplementary file 1 [file ECE3-8-11122-s001.docx]

Appendix 1. Mean (SE) resource characteristics of New England cottontail habitat delineated as early (*n*=9), mid (*n*=16), or late (*n*=12) successional or persistent shrubland (*n*=11).in New York, 2013-2016. Data obtained from plot surveys (*n=*1,191) across 50 m grids conducted within aforementioned sites.

| Variable | Early | Mid | Late | Persistent |
| --- | --- | --- | --- | --- |
| Proportion persistent canopy closure^a^ | 0.17 (0.02) | 0.47 (0.05) | 0.61 (0.05) | 0.48 (0.03) |
| Proportion seasonal canopy closure^a^ | 0.43 (0.07) | 0.63 (0.03) | 0.89 (0.02) | 0.83 (0.04) |
| Mean maximum forb height (m)^b^ | 0.46 (0.05) | 0.30 (0.06) | 0.10 (0.02) | 0.24 (0.05) |
| Shrub height (m)^b^ | 0.48 (0.10) | 0.53 (0.07) | 0.52 (0.05) | 0.41 (0.04) |
| Forb cover^c^ | 0.32 (0.07) | 0.24 (0.07) | 0.08 (0.03) | 0.20 (0.06) |
| Shrub cover^c^ | 0.44 (0.09) | 0.54 (0.07) | 0.53 (0.06) | 0.37 (0.05) |
| Graminoid cover^c^ | 0.27 (0.07) | 0.09 (0.04) | 0.06 (0.02) | 0.05 (0.02) |
| Japanese barberry stems per 10 m^2d^ | 5.11 (2.63) | 19.33 (6.47) | 25.74 (4.87) | 7.14 (5.35) |
| Multiflora rose stems per 10 m^2d^ | 14.07 (9.5) | 15.76 (7.57) | 5.63 (1.95) | 4.24 (1.12) |
| Palatable native stems per 10 m^2d^ | 10.7 (3.75) | 5.70 (1.84) | 2.72 (0.75) | 6.42 (1.92) |

^a^ Assessed using spherical densitometer at a height of 1 m

^b^ Assessed as average maximum height of vegetation

^c^ Assessed as the proportion of each cover type in a 1 m plot at each point

^d^ Assessed by counting woody stems <7.5 cm dbh of each species in a 1 m x 10 m plot at a height of 0.5m

Appendix 2. Sample sizes of locations of New England (*Sylvilagus transitionalis*, NEC) and eastern cottontails (*Sylvilagus floridanus*, EC) by season (leaf-off and leaf-on), species, and eastern cottontail prevalence categories (less, more) for different ratios of eastern: New England cottontails. We used a ratio of 1:5 to categorize EC prevalence. Assessing resource selection for eastern cottontails where they were less prevalent (dashed bars) was not a target of this study and data were insufficient for analyses.

Appendix 3. Sample sizes of number of New England (*Sylvilagus transitionalis*, NEC) and eastern cottontails (*Sylvilagus floridanus*, EC) by season (leaf-off and leaf-on), species, and eastern cottontail prevalence categories (less, more) for different ratios of eastern: New England cottontails. We used a ratio of 1:5 to categorize EC prevalence. Assessing resource selection for eastern cottontails where they were less prevalent (dashed bars) was not a target of this study and data were insufficient for analyses.Appendix 4. List plants identified and considered as native palatable vegetation for New England cottontails in New York 2013-2016. Palatability determined from feeding trials of Pringle (1960)^a^.

| Scientific name | Common name |
| --- | --- |
| *Acer pensylvanicum* | Striped maple |
| *Acer rubrum* | Red Maple |
| *Acer saccharum* | Sugar Maple |
| *Acer saccharum* | Sugar Maple |
| *Acer* spp. | Maple spp. |
| *Betula lenta* | Black Birch |
| *Cornus alternifolia* | Alternate-leaved dogwood |
| *Cornus amomum* | Silky Dogwood |
| *Cornus florida* | Flowering dogwood |
| *Cornus racemosa* | Gray Dogwood |
| *Cornus sericea* | Red Osier Dogwood |
| *Cornus* spp. | Dogwood Spp. |
| *Fagus grandifolia* | American beech |
| *Gaultheria procumbens* | Wintergreen |
| *Prunus serotina* | Black Cherry |
| *Prunus virginiana* | Choke Cherry |
| *Quercus* spp. | Oak spp. |
| *Rhus glabra* | Smooth sumac |
| *Rhus* spp. | Sumac spp. |
| *Rubus occidentalis* | Black-capped raspberry |
| *Rubus pensilvanicus* | Pennsylvania blackberry |
| *Rubus* spp. | Raspberry |
| *Sassafras albidum* | Sassafrass |
| *Vaccinium angustifolium* | Lowbush blueberry |
| *Vaccinium corymbosum* | Highbush blueberry |
| *Vaccinium* spp. |  |
| *Viburnum lentago* | Nannyberry |
| *Viburnum spp.* | Viburnum spp. |
| *Vitis* Spp. | Grape spp. |

**^a^** Pringle, L.P. (1960) A study of the biology and ecology of the New England cottontail (S*ylvilagus transitionalis*) in Massachusetts. M.S. thesis, University of Massachusetts, Amherst, MA, USA.

Appendix 5. Number of New England and Eastern cottontail individuals monitored at sites in the Hudson Valley, New York between December 2013 and July 2016 for resource selection analyses.

| Site | Eastern Cottontail | New England cottontail |
| --- | --- | --- |
| 1 | 5 | 11 |
| 2 | 14 | 1 |
| 3 | 4 | 10 |
| 4 | 1 | 1 |
| 5 | 8 | 0 |
| 6 | 1 | 0 |
| 7 | 0 | 1 |
| 8 | 2 | 6 |
| 9 | 10 | 4 |
| 10 | 1 | 6 |
| 11 | 0 | 5 |
| 12 | 12 | 4 |
| 13 | 1 | 2 |
| 14 | 5 | 10 |
| 15 | 4 | 11 |
| 16 | 1 | 8 |

Appendix 6. Mean (SE) vegetation characteristics of shrubland patches where the ratio of eastern to New England cottontails was greater than 1:6 among known-alive individuals and (more prevalent) and equal to or less than 1:6 (less prevalent) within sites designated as early (*n*=9), mid (*n*=16), and late successional shubland (*n*=12), and persistent shrubland (*n*=11) patches in New York, 2013-2016.

|  | Early | |  | Mid | |  | Late | |  | Persistent | |
| --- | --- | --- | --- | --- | --- | --- | --- | --- | --- | --- | --- |
| Variable | Less prevalent | More prevalent |  | Less prevalent | More prevalent |  | Less prevalent | more prevalent |  | Less prevalent | More prevalent |
| Persistent canopy^a^ | 0.13 (0.03) | 0.18 (0.02) |  | 0.53 (0.06) | 0.41 (0.05) |  | 0.70 (0.07) | 0.57 (0.05) |  | 0.49 (0.03) | 0.47 (0.03) |
| Seasonal canopy | 0.56 (0.17) | 0.45 (0.07) |  | 0.68 (0.07) | 0.62 (0.03) |  | 0.90 (0.02) | 0.87 (0.03) |  | 0.87 (0.03) | 0.84 (0.04) |
| Forb height^b^ | 0.42 (0.07) | 0.47 (0.05) |  | 0.32 (0.08) | 0.30 (0.06) |  | 0.08 (0.03) | 0.12 (0.03) |  | 0.18 (0.04) | 0.26 (0.06) |
| Shrub height | 0.42 (0.10) | 0.52 (0.09) |  | 0.50 (0.10) | 0.49 (0.07) |  | 0.44 (0.09) | 0.56 (0.06) |  | 0.46 (0.05) | 0.45 (0.03) |
| Forb cover^c^ | 0.36 (0.15) | 0.34 (0.07) |  | 0.23 (0.05) | 0.19 (0.03) |  | 0.07 (0.03) | 0.10 (0.03) |  | 0.14 (0.02) | 0.23 (0.07) |
| Shrub cover | 0.32 (0.13) | 0.48 (0.08) |  | 0.54 (0.08) | 0.56 (0.06) |  | 0.47 (0.10) | 0.56 (0.06) |  | 0.44 (0.05) | 0.40 (0.05) |
| Grass cover | 0.38 (0.02) | 0.23 (0.06) |  | 0.19 (0.09) | 0.11 (0.04) |  | 0.09 (0.03) | 0.06 (0.02) |  | 0.03 (0.01) | 0.05 (0.02) |
| Barberry stems^d^ | 10.8 (6.91) | 5.51 (2.84) |  | 15.54 (13.53) | 13.21 (5.23) |  | 16.33 (8.44) | 25.29 (6.04) |  | 12.85 (9.51) | 8.58 (6.50) |
| Rose Stems | 5.47 (3.25) | 15.50 (10.60) |  | 21.75 (10.95) | 11.63 (4.62) |  | 10.30 (4.16) | 5.78 (2.24) |  | 3.12 (1.34) | 3.78 (1.22) |
| Palatable stems | 9.20 (8.90) | 12.03 (3.97) |  | 4.23 (1.49) | 6.11 (1.94) |  | 2.06 (0.57) | 3.11 (0.90) |  | 8.20 (3.36) | 7.40 (2.22) |

^a^Proportion canopy closure

^b^In meters

^c^Proportion cover

^d^Stems per 10 m^2^

Appendix 7. Posterior summaries (mean, SD) for hyperparameters of the mixed-effects conditional logistic regression resource selection models with random effects of individual for New England cottontails (*Sylvilagus transitionalis*) by eastern cottontail (*S. floridanus*) prevalence^a^ by season in New York, 2013 - 2016.

|  |  | Leaf-off season | |  | Leaf-on season | |
| --- | --- | --- | --- | --- | --- | --- |
| Variable | Parameter | Less prevalent | More prevalent |  | Less prevalent | More prevalent |
| Persistent Canopy | β^b^ | 13.29 (2.51) | 1.66 (0.51) |  | -0.26 (1.00) | 0.99 (0.51) |
|  | σ | 8.82 (0.91) | 2.28 (0.5) |  | 3.87 (1.06) | 1.49 (0.57) |
| Persistent Canopy^2^ | β | -10.93 (2.17) |  |  |  |  |
|  | σ | 7.31 (1.44) |  |  |  |  |
| Seasonal Canopy | β |  |  |  | 9.04 (2.09) | -0.76 (0.50) |
|  | σ |  |  |  | 5.07 (1.34) | 1.25 (0.57) |
| Seasonal Canopy^2^ | β |  |  |  | -5.84 (1.42) |  |
|  | σ |  |  |  | 2.59 (1.14) |  |
| Forb Height | β |  |  |  | -1.33 (0.96) | 0 (0.01) |
|  | σ |  |  |  | 0.01 (0.01) | 0.02 (0.01) |
| Shrub Height | β | 2.1 (1.55) | 1.44 (0.55) |  | 1 (0.7) | 0.03 (0.47) |
|  | σ | 5.07 (1.18) | 2.33 (0.58) |  | 2.43 (0.77) | 1.21 (0.58) |
| Shrub Height^2^ | β | -5.07 (1.33) |  |  |  |  |
|  | σ | 2.6 (1.16) |  |  |  |  |
| Forb Cover | β |  |  |  | -1.33 (0.96) | 1.97 (1) |
|  | σ |  |  |  | 2.58 (0.95) | 1.45 (0.85) |
| Forb Cover^2^ | β |  |  |  |  | -4.04 (1.36) |
|  | σ |  |  |  |  | 1.21 (0.89) |
| Shrub Cover | β | 5.34 (1.49) | 4.64 (1.08) |  | 0.02 (0.57) | 5.53 (1.09) |
|  | σ | 4.29 (1.07) | 2.74 (0.8) |  | 1.04 (0.69) | 1.03 (0.65) |
| Shrub Cover^2^ | β | -5.07 (1.33) | -4.70 (1.12) |  |  | -4.39 (1.01) |
|  | σ | 2.21 (1.24) | 2.61 (0.9) |  |  | 1.00 (0.58) |
| Grass Cover | β |  |  |  | 5.01 (1.22) | -1.5 (0.87) |
|  | σ |  |  |  | 1.52 (1.08) | 2.87 (0.94) |
| Grass Cover^2^ | β |  |  |  | -5.34 (2.4) |  |
|  | σ |  |  |  | 5.18 (1.88) |  |
| Japanese Barberry | β | 1.01 (1.02) | 4.83 (1.19) |  | -0.09 (0.64) | 0.8 (0.61) |
|  | σ | 3.71 (1.37) | 3.33 (2.06) |  | 1.38 (0.81) | 0.91 (0.71) |
| Japanese Barberry^2^ | β |  | -5.45 (1.86) |  |  |  |
|  | σ |  | 3.51 (1.81) |  |  |  |
| Multiflora Rose | β | -2.91 (2.73) | -4.4 (1.62) |  | 4.65 (2.14) | -1.08 (1.54) |
|  | σ | 8.51 (1.22) | 4.7 (1.6) |  | 4.35 (2.47) | 3.83 (1.94) |
| Native Palatable | β | -2.94 (2.69) | 2.31 (1.43) |  | -5.88 (2.76) | 1.77 (1.3) |
|  | σ | 8.43 (1.25) | 4.85 (1.74) |  | 6.42 (2.39) | 3.16 (1.62) |

^a^ “More prevalent” = eastern cottontails comprise >1 in 6 known-alive cottontails

^b^Coefficients not comparable among variables

Appendix 8. Posterior summaries (mean, SD) for hyperparameters of the conditional logistic regression resource selection models with random effect of individual for eastern cottontails (*Sylvilagus floridanus*) at sites where they were more prevalent than 1 in 6 known-alive cottontails, by season in New York, 2013 - 2016.

| Variable | Parameter | Leaf-off Season | Leaf-on Season |
| --- | --- | --- | --- |
| Persistent Canopy | β^a^ | 1.43 (1.02) | -1.64 (0.72) |
|  | σ | 3.15 (0.56) | 3.54 (0.68) |
| Persistent Canopy^2^ | β | -2.5 (1.01) |  |
|  | σ | 0.91 (0.68) |  |
| Seasonal Canopy | β |  | 2.52 (1.05) |
|  | σ |  | 1.46 (0.65) |
| Seasonal Canopy^2^ | β |  | -3.59 (0.86) |
|  | σ |  | 0.75 (0.5) |
| Forb Height | β |  | 0.00 (0.00) |
|  | σ |  | 0.01 (0.00) |
| Shrub Height | β | 0.13 (0.37) | 0.04 (0.44) |
|  | σ | 1.88 (0.38) | 1.81 (0.46) |
| Forb Cover | β |  | 2.7 (0.95) |
|  | σ |  | 2.28 (0.82) |
| Forb Cover2 | β |  | -3.89 (1.04) |
|  | σ |  | 1.18 (0.85) |
| Shrub Cover | β | 5.53 (0.74) | 2.67 (0.95) |
|  | σ | 1.41 (0.59) | 1.06 (0.63) |
| Shrub Cover2 | β | -5.34 (0.72) | -2.51 (0.88) |
|  | σ | 1.76 (0.52) | 1.64 (0.59) |
| Grass Cover | β |  | 0.84 (0.91) |
|  | σ |  | 2.1 (0.98) |
| Grass Cover2 | β |  | -3.4 (1.5) |
|  | σ |  | 3.47 (1.38) |
| Japanese Barberry | β | 0.79 (0.49) | 0.69 (0.61) |
|  | σ | 1.57 (0.62) | 1.57 (0.87) |
| Multiflora Rose | β | -0.97 (1.15) | -1.96 (1.56) |
|  | σ | 4.44 (1.76) | 5.99 (1.97) |
| Native Palatable | β | 1.96 (1) | 1.33 (1.66) |
|  | σ | 4.26 (1.37) | 7.88 (1.22) |

^a^Coefficient means not comparable among variables


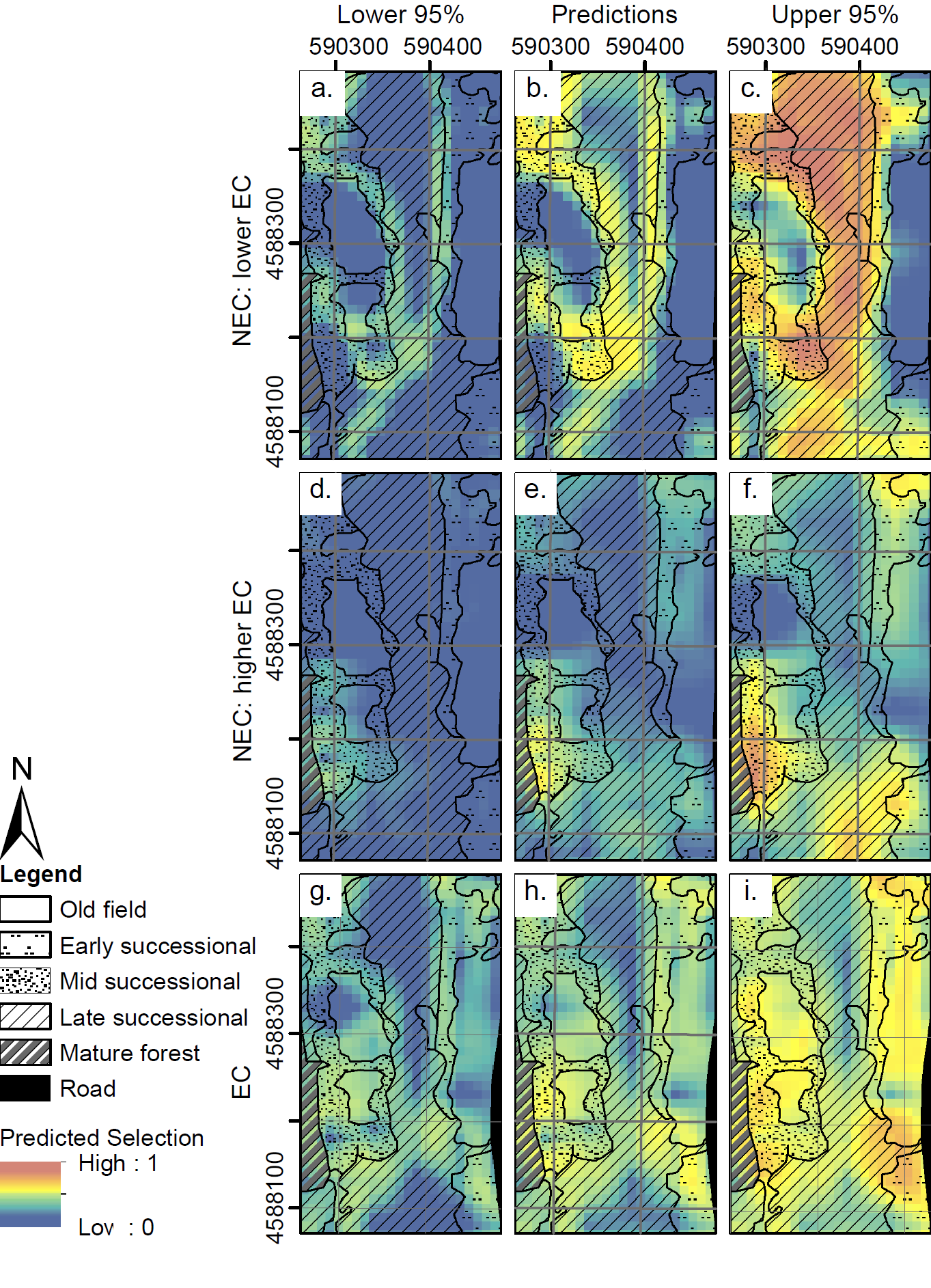


Appendix 9. Example of patch-level cover types and leaf-off predicted a. lower 95% predictions interval, b. predictions, and c. upper 95% prediction interval for resource selection of New England cottontails (NEC) where eastern cottontails (EC) were less prevalent; d. lower 95% prediction intervals, e. predictions, and f. upper 95% prediction intervals for resource selection of New England cottontails where eastern cottontails were more prevalent; and g. lower 95% predictions, h. predictions, and i. upper 95% prediction interval for resource selection of eastern cottontails.


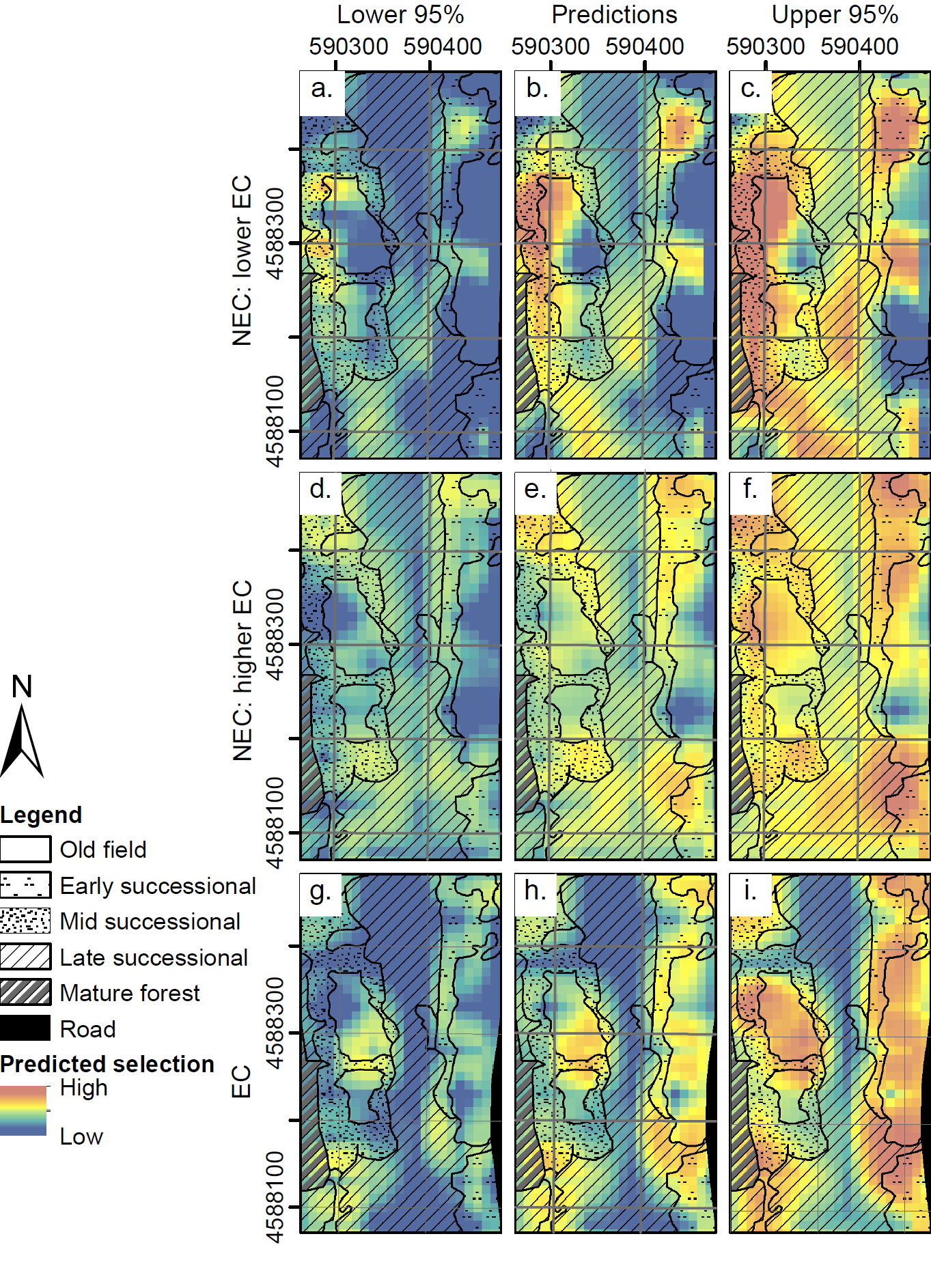


Appendix 10. Example of patch-level cover types and leaf-on predicted a. lower 95% predictions interval, b. predictions, and c. upper 95% prediction interval for resource selection of New England cottontails (NEC) where eastern cottontails (EC) were less prevalent; d. lower 95% prediction intervals, e. predictions, and f. upper 95% prediction intervals for resource selection of New England cottontails where eastern cottontails were more prevalent; and g. lower 95% predictions, h. predictions, and i. upper 95% prediction interval for resource selection of eastern cottontails.


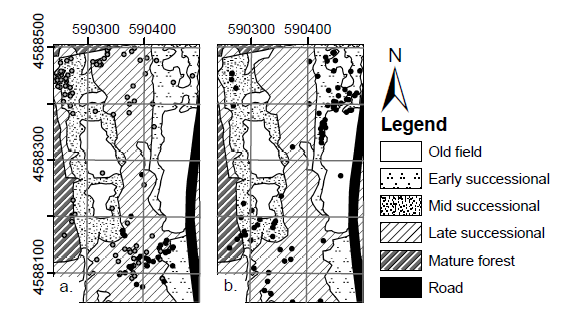


Appendix 11. Example of a site where the prevalence of eastern cottontails changed from less prevalent to more prevalent between years, depicting a. locations of New England cottontails when eastern cottontails were less prevalent (gray circles, 7 individuals) and when eastern cottontails were more prevalent (black circles, 2 individuals), and b. locations of eastern cottontails where they were more prevalent (black circles, 4 individuals).
